# GMO Food Labels Do Not Affect College Student Food Selection, Despite Negative Attitudes towards GMOs

**DOI:** 10.3390/ijerph18041761

**Published:** 2021-02-11

**Authors:** Katrina Oselinsky, Ashlie Johnson, Pamela Lundeberg, Abby Johnson Holm, Megan Mueller, Dan J. Graham

**Affiliations:** 1 Department of Psychology, Colorado State University, Fort Collins, CO 80523, USA; ashlie.johnson@colostate.edu (A.J.); pamela.lundeberg@colostate.edu (P.L.); abby.johnson@colostate.edu (A.J.H.); dan.graham@colostate.edu (D.J.G.); 2 Department of Food Science and Human Nutrition, Colorado State University, Fort Collins, CO 80523, USA; megan.mueller@colostate.edu

**Keywords:** GMO, food labels, food choice, attitude–behavior gap

## Abstract

US Public Law 114–216 dictates that food producers in the United States of America will be required to label foods containing genetically modified organisms (GMOs) starting in 2022; however, there is little empirical evidence demonstrating how U.S. consumers would use food labels that indicate the presence or absence of GMOs. The aim of this two-phase study was to determine how attitudes towards GMOs relate to food choices and how labels indicating the presence or absence of GMOs differentially impact choices among college students—the age group which values transparent food labeling more than any other. Participants (*n* = 434) made yes/no choices for each of 64 foods. In both phases of the study, participants were randomly assigned to seeing GMO Free labels, contains GMOs labels, or no GMO labels. Across the two phases, 85% of participants reported believing that GMOs were at least somewhat dangerous to health (42% believed GMOs to be dangerous), yet in both studies, although eye-tracking data verified that participants attended to the GMO labels, these labels did not significantly affect food choices. Although college consumers may believe GMOs to be dangerous, their food choices do not reflect this belief.

## 1. Introduction

Public Law 114–216 dictates that, beginning 1 January 2022, U.S. food producers must label food products that are or may be bioengineered. This law defines bioengineered foods as “those that contain detectable genetic material that has been modified through certain lab techniques and cannot be created through conventional breeding or found in nature” [1]. Although this law has already been implemented in some food manufacturing settings, the introduction of Public Law 114–216 has sparked concerns that the new labels will be used by consumers to discriminate against genetically modified (GM) products [2,3,4]. Though this is a widespread concern, few studies have evaluated whether GM disclosures actually affect consumer choice. In order to estimate the impact of Public Law 114–216 on consumer choice, research assessing the impact of nutrition labels, specifically those related to genetic modification, must be evaluated. The aim of the present study is to assess how labels indicating the presence or absence of genetic modification impact college students’ food selection decisions. Although data collection for the present study occurred prior to the passing of the Public Law 114–216, this work can be used to inform how these forthcoming labels might impact college-aged consumers.

While the estimated percent of consumers who accept or oppose genetically modified organisms (GMOs) fluctuates across years and populations, overall, a sizeable proportion of consumers (estimates range from 15 to 75%) hold negative views towards GM products, with European consumers being less accepting than their US counterparts [5,6,7]. A meta-analysis conducted by Lusk et al. [8] concluded that, across Europe, Asia, and Oceania, consumers were willing to pay a premium of 23–42% more for non-GM products vs. modified alternatives. Recent research by Ardebili and Rickertsen [9] concluded that consumers remain willing to pay a similar premium for non-GM food today. Although scientific evidence indicates that GM foods pose no human or environmental health risks, the public perception of GMOs diverges significantly. In the US, upwards of 75% of consumers are concerned about the perceived health and environmental risks of GM foods; 70% of consumers believe that consuming GM food is dangerous and 51% of Americans perceive GMOs to be worse for health than non-GM alternatives [6,10,11,12]. Although attitude has been successfully used to predict behavior within many health decision contexts [13,14,15,16,17], attitude alone may be insufficient for predicting consumer purchasing behavior with regard to GM-labeled products.

The attitude–behavior gap (sometimes referred to as the intention–behavior gap) identifies an incongruence between self-reported attitudes and subsequent behaviors [18]. A compelling example of this gap within the food purchasing/consumption literature comes from the purchasing of organic products [19,20,21,22,23]. Hidalgo-Baz et al. [24], for example, ascertained that a wide gap exists between consumers’ stated preference for organic products (which is high) and their actual purchasing behavior (which is low). While Hidalgo-Baz et al. [24] posit that many factors could be contributing to this divide, the result is clear: Attitude alone is not enough to predict actual purchasing behavior for organic products. Given the ambiguity regarding the relationship between attitudes and food purchasing decisions, particularly for foods characterized by a potentially emotion-laden descriptor such as “organic” or “GM”, one cannot assume that a negatively expressed attitude towards GM food products will translate into avoidant behavior. Just as consumers report a preference for organic products and then fail to purchase these products, consumers may report disliking GM products and then, nevertheless, purchase them. As such, it is worth investigating how self-reported attitudes and both types of GMO labels (i.e., contains GMOs and GMO free) impact consumers’ food choices.

Research by Roe and Teisl [25] found that labels indicating the presence of genetic modification were viewed as being more credible than labels indicating the absence of genetic modification, but that the latter labels were more likely to be rated as providing sufficient information to make an informed food selection decision. Additionally, to assess the impact of GM labels on consumers’ food choices, research by Yeh et al. [2] exposed participants to different label sequences. In this study, 1306 subjects participated in an online survey in which they were shown fruits and vegetables containing a label stating the presence or absence of genetic modification or no GM label. The order in which labels were displayed varied across participants. The results indicate that in an attempt to avoid products that contain GMOs (as indicated by a “Genetically Modified” label), the consumer demand for unlabeled products increased. The authors assert that when faced with a label clearly indicating the presence of genetic modification, consumers will no longer differentiate between products stating they do not contain GMOs and unlabeled products [2]. Although this study provides insight into how consumers may respond to Public Law 114–216, the authors note that their study only utilized fresh produce (a category of foods which is not subject to other labeling laws, such as those mandating a nutrition facts panel, and for which GM versions are not widely available). Additionally, the ages of the participants were restricted (to 21+), eliminating a sizeable subset of Generation Z, including most college students at the age when they transition from living with parents to living, and making food choices, independently.

As the U.S. population ages, millennials (those born between 1981 and 1996) and Generation Z (those born after 1996) are becoming the majority demographic within the food purchasing market. Hartman [26] estimates that together, these generations account for 51% of the U.S. population and by 2030, will be the largest global cohort [27]. However, much of the existing literature examining nutrition label use has focused on adult populations (mean participant age = 40–50 years) [28,29]. Despite often being overlooked, emerging adulthood is a critical time during which many individuals develop lasting health behavior patterns [30]. It is therefore important to understand how college students utilize GM labels, as the habits they develop now will likely remain stable for decades to come [28]. Additionally, although general trends show that both millennials and Generation Z share certain food-related values (i.e., consumers from these generations prefer fresh, organic options), Generation Z is the age group which cares most about healthy and organic food products and places a higher value than any other group on transparent labeling [26,31]. Due to this prioritization of consuming foods perceived to be healthful, it is likely that members of Generation Z (a group which includes most U.S. university/college students) will be the most interested in and sensitive to labels depicting the presence or absence of genetic modification.

Although consumer food choice will be highly influential in determining how food manufacturers respond to the forthcoming mandatory GM labeling law, scant research has focused on how consumers utilize GM labels when making food choices. Much of the current literature has used consumer attitudes as a means to predict subsequent purchasing behavior; however, research indicates that attitude alone is not sufficient for predicting food selection. Lastly, although some work has evaluated the impact of GM label introduction on consumer demand for fruits and vegetables [2], more work is needed to identify how consumers will respond to the new labeling law across a variety of food products, especially those most likely to receive the new label. To fill this gap in the literature, the present study assesses the influence of labels indicating the presence or absence of GMOs on college consumers’ food choices, as well as the interaction between college consumers’ self-reported concerns about GMOs and food selection.

### Hypotheses

The research team hypothesized the following:

1. The number of foods selected will be lower for products with contains GMOs labels compared to unlabeled products
  a Rationale: Due to the widespread negative attitudes towards GM products [11,12], contains GMOs labels are expected to discourage individuals from purchasing products labeled like this;
2. The number of foods selected will be higher for products with GMO free labels compared to those with no labels.
  a Rationale: Conversely, the research team predicted that the presence of GMO free labels would have the opposite effect. Since a large proportion of consumers hold negative attitudes towards GMOs, labels stating the absence of genetic modification would be expected to encourage purchasing among those who are averse to such practices;
3. Risk perception will interact with the label type such that, as the perceived GMO risk increases, the number of foods selected for items with contains GMOs labels will decrease and the number of foods selected labeled GMO free will increase.
  a Rationale: For those who perceive a higher risk associated with GMO consumption, labels indicating the presence of genetic modification will prompt participants to avoid those products, while GMO free labels will signal safety and encourage consumer choice.

## 2. Materials and Methods

### 2.1. Study Design

This study consists of a computerized choice experiment conducted in two phases. Using a between-subjects design, participants were randomly assigned to one of three conditions: (1) GMO free labels; (2) contains GMOs labels; or (3) the control (no GMO-related labels). In all three conditions, participants saw each food product’s relevant nutrition facts label, a photograph of the food without identifiable brand packaging, a product description including health claims and front-of-package information, the product’s price (reflecting the purchase price of the items at the time they were acquired for the experiment), and a list of the product’s ingredients (see Appendix B for a sample simulation trial for each condition). Participants were instructed to indicate whether they would purchase each food based on the information provided. In both phases, the control condition was the same: Participants saw 64 food products, none of which had GMO labels. For these participants, GMOs were not mentioned at all during the food choice task. There were differences in the GMO free and contains GMO conditions between the two study phases. As phase 1 was designed to mimic a real-world food choice task (e.g., shopping in a grocery store, where products containing GMOs—here labeled with a contains GMOs label—could also not contain GMOs, and so would not be labeled as GMO free), half of the food products (i.e., 32 foods) were assigned a GMO free label, and the other half of the food products were assigned a contains GMOs label. In the GMO free condition, half the foods contained labels declaring the products to be GMO free, while the other 32 foods presented to participants in this condition had no GMO-related labels. In the contains GMOs condition, the opposite 32 foods (those not labeled GMO free in the GMO free condition) were given contains GMOs labels, while the other 32 foods had no GMO labels.

In order to further assess the role of the GMO labels in participants’ food choices, we followed up with phase 2 of the study, where the foods that had GMO labels were constant. In this experiment, the same food items received labels in the contains GMOs and GMO free conditions, in order to eliminate the possibility that participants would report that they would or would not purchase a given item, regardless of the GMO label shown. For example, some participants will never select cheese puffs, regardless of whether this product has a GMO label—that specific product will not appeal to all consumers. Therefore, the real-world situation in which a food does not simultaneously contain and not contain GMOs (i.e., the situation tested in phase 1) was supplemented with phase 2′s controlled situation in which the foods that received labels were kept constant, in order to evaluate different labels’ impacts on food choice for the very same products.

To evaluate whether GMO labels were salient for participants, the research team utilized eye-tracking technology to determine the extent to which participants were viewing GMO labels (or, for control participants, the blank area of the screen where GMO labels were presented in the other two conditions). We further describe the methods and results for both phases of the study below.

### 2.2. Participants 

In phase 1, data were collected in the fall of 2015. A convenience sample of 203 (GMO free *n* = 70; contains GMOs *n* = 63; control = 70) students from a large university in the Western US participated in this phase. In phase 2, an additional convenience sample of 231 (GMO free *n* = 61; contains GMOs *n* = 128; control = 42) students were recruited in the spring of 2016. Those who participated in phase 1 were unable to participate in phase 2, rendering the samples distinct from one another. Participants were recruited using a university-based online recruitment system for undergraduate students enrolled in introductory psychology courses. Through this system, participants select studies they would like to complete based on a brief description of the study and its participation requirements. All data for both phases of the study were collected in a basement laboratory of an on-campus university building in a dedicated research laboratory space, approximately 3 m by 4 m in size. The participant and experimenter were the only two people in the space during each 60-min laboratory visit. Participants were compensated for their participation via course credits. Data for one participant in phase 1 and two participants in phase 2 were incomplete and were removed from analyses. To be eligible to participate, students needed accurate vision (the ability to read study materials on a computer monitor at a distance of 55 cm) or vision corrected via soft contact lenses. Those with glasses or hard contact lenses were excluded due to incompatibility with eye-tracking equipment used to assess visual attention. Participants recruited in both phases were demographically similar. The majority of participants were female and white and the mean age was around 19 years (Table 1). Both participant samples are representative of the state’s population in terms of race/ethnicity [32]. Additionally, within both samples, the majority of participants were within the age range to be considered members of Generation Z, which is a highly influential, yet understudied population within the food selection literature [26,27].

The research team obtained approval from its institutional review board prior to data collection, and all participants were required to sign an informed consent document before engaging in study activities.

### 2.3. Procedures

Data collection for phase one began in September of 2015 and concluded in December. Phase two data collection began in January of 2016 and ended in May of the same year. Participants were first given an informed consent document that provided a short explanation of the study and detailed what tasks they would be asked to complete. After consenting, an eye-tracking camera was calibrated to their dominant eye. The eye-tracking camera used in this research was the EyeLink 1000 (SR Research, Ottawa, Ontario, Canada). Following consent and calibration, participants were randomly assigned to a labeling condition. In both phases, participants were presented with images of 64 different foods from 15 food categories, including cookies, ice creams, crackers, nuts, chips, salty snacks (pretzels, cheese puffs, and rice cakes), yogurts, soups, cereals, meats, pizzas, canned fruit, canned vegetables, frozen fruit, and frozen vegetables in a computerized choice experiment (a complete list of all products and their GMO labels by study phase and condition can be found in Appendix C). Only foods that were packaged and therefore contained the nutrition facts panel were included. Participants were asked to indicate whether they would purchase each product in a computerized food choice task consisting of 64 trials. In all conditions, participants were asked to consider the available information and indicate whether they would purchase the food by selecting “I would consider buying this product,” “I would NOT consider buying this product,” or “Not Applicable (I would not eat this)” (participants were instructed to only select this last option for any items they could not eat due to food allergies/dietary restrictions). The research team utilized eye-tracking technology to determine the extent to which participants were viewing GMO labels (or the blank area of the screen where a GMO label was not included in the control condition). After completing all 64 selection trials, participants provided demographic information and completed the post-task questionnaire described in the Measures section.

### 2.4. Measures 

#### 2.4.1. Outcome Variable: Food Choice

In both study phases, participants were instructed to use the information provided for each of the 64 different food products to determine if they would buy the product or not. Food choice was conceptualized as a summation of the number of ‘would buy’ responses per participant across the 64 trials.

#### 2.4.2. Covariates: Post-Food-Selection Questionnaire

Participants in both phases completed a short survey after the food choice task. Participants self-reported demographic information, including sex, age, race, and ethnicity. Lastly, participants were asked to self-report their perception of the study’s purpose, to define the term “GMO” to the best of their ability (open-ended), and to report how dangerous they believed it was to consume GMOs on a 4-point scale ranging from not at all (0) to very (3).

#### 2.4.3. Eye-Tracking Data

As a validity check, eye-tracking data (i.e., milliseconds of visual attention devoted to the GMO label region on the screen) were used to evaluate whether participants spent some portion of time looking at the GMO labels.

### 2.5. Analyses

Analyses were conducted in 2019 using RStudio Statistical Software version 3.6.1 (RStudio Team, Boston, MA, USA.) [33]. Multiple linear regression analysis (MLR) with categorical predictors was used to determine the impact of the label condition (control/no label, GMO free label, and contains GMOs label) on food choice. Due to the multiple comparisons made (control vs. GMO free, control vs. contains GMOs, and GMO free vs. contains GMOs), the alpha was subjected to a Bonferroni correction, resulting in a more conservative significance level (*p* < 0.017). Given the differences in study design, separate models were run for the two phases of the study.

A Shapiro Test for normality indicated that the outcome variable (the total number of items selected) followed a normal distribution (S1 *p* = 0.516). An MLR model with all 64 food items was created, and the ‘relevel’ function was used to change the reference group (control to GMO free), in order to make all possible between-group comparisons. Additional sensitivity analyses were conducted to evaluate whether looking only at the 32 items that had labels impacted participants’ food selection decisions (see Appendix A).

Previous research has shown that attitude can successfully predict subsequent behavior [13,14,15,16,17]. When applied to the current research, this line of work implies that a negative attitude towards GM food products should translate into the behavior of avoiding such products. To test this theory, interaction models were run to assess whether the perceived GMO risk impacted food choice. The interaction term “risk” was applied to each condition and was included in both MLR models. Bonferroni Outlier Tests produced no values outside the anticipated range.

## 3. Results

Across the two studies, 85% of participants reported believing that GMOs were at least somewhat dangerous to consume (42% believed GMOs to be very dangerous). The eye-tracking data confirmed that all participants in the labeling conditions spent some portion of time (defined as fixating on the label at least once) looking at the GMO labels. Those in the control condition spent no meaningful time (<10 milliseconds) looking at the “GMO label” area as no label was present.

The results of the MLR model indicated no significant differences in food choice based on label type or demographic characteristics (see Table 2 and Table 3). When comparing the contains GMOs to the control condition, on average, participants selected 3.054 fewer foods when shown a contains GMOs label as opposed to no label (31.889 for contains GMOs vs. 34.943 for control, see Table 2); however, this difference had a *p* = 0.048, which did not meet the Bonferroni-corrected level for statistical significance.

The “relevel” function was used to reassign the reference group in order to compare the GMO free to the contains GMOs condition. The results showed that the participants in the contains GMOs condition selected an average of 3.368 fewer food items than those in the GMO free condition (*p* = 0.029, see Table 4). Due to the Bonferroni correction, this result was also not statistically significant. Finally, we found no differences in the results in our sensitivity analyses (Appendix A).

The results indicate that the GMO labels themselves did not have a significant impact on participants’ food choice.

**Figure 1:**
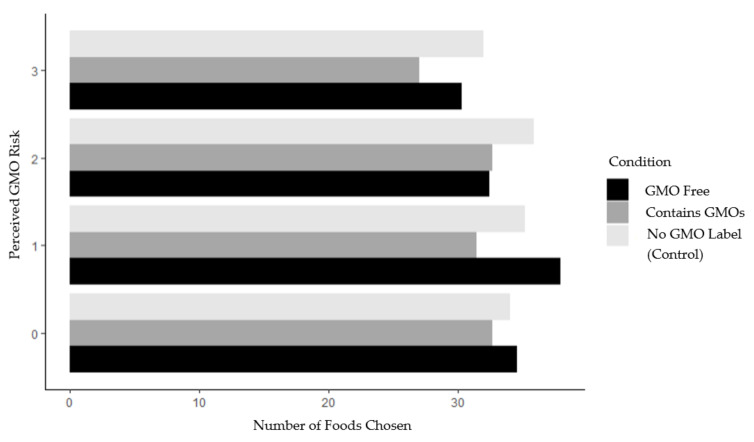
Phase 1 number of foods selected (of 64 possible) by perceived GMO risk and study condition. *Note*: GMO risk ranged from zero (no perceived danger associated with consuming foods containing GMOs) to 3 (a great deal of perceived danger from consuming GMOs). GMO free = 32 of 64 products in this condition were labeled GMO free; contains GMOs = 32 of 64 products in this condition (those not labeled GMO free in the GMO free condition) labeled contains GMOs.

Interaction models were evaluated to examine the differential effect of perceived GMO risk on label type. A moderation model was estimated with risk regressed on the categorical indicators of condition and their interaction. The interaction term was not statistically significant for any condition, indicating that the perceived level of danger self-reported by the participants did not have any impact on their food choice (see Figure 1). We further tested whether there was a threshold at which the level of perceived risk had an impact on food choice. No such level was identified.

**Figure 2:**
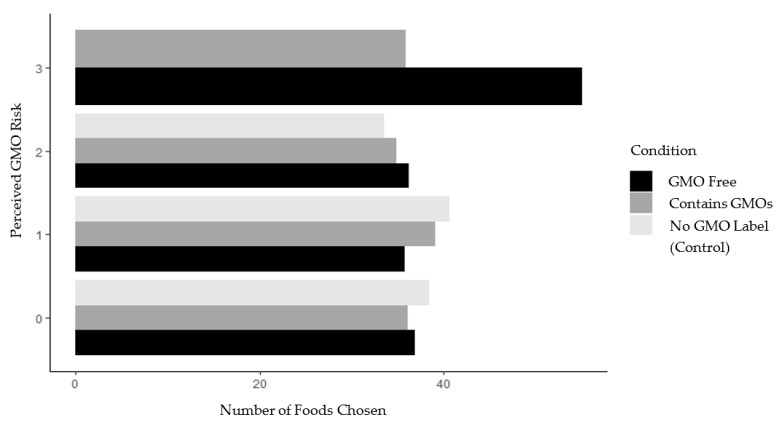
Study 2 number of foods selected (of 64 possible) by perceived GMO risk and study condition. *Note*. GMO risk ranged from zero (no perceived danger associated with consuming foods containing GMOs) to 3 (a great deal of perceived danger from consuming GMOs). GMO free = 32 of 64 products in this condition were labeled GMO free; contains GMOs = 32 of 64 products in this condition (the same products labeled GMO free in the GMO free condition) labeled contains GMOs.

Phase 2 of the study generated similar results. There were no significant differences in food choice between conditions. Participants in the control condition selected an average of 37.690 items (see Table 4). Those in the GMO free condition and the contains GMOs condition selected an average of 36.328 and 36.719 foods, respectively (see Table 4). The eye-tracking data indicated that, as in phase 1, phase 2 participants in the GMO free and contains GMOs conditions did view the GMO labels (participants in these conditions looked at the GMO label an average of 2.270 times), whereas those in the control condition did not view this blank, label-less region of the screen at all. Since the same food items received labels, the research team was able to compare the impact of two different labels on the same foods. No significant differences were found in participants’ food choice based on label type or demographic characteristics (see Table 5 and Table 6). An interaction model was run with self-reported perceptions of GMO risk and the results were consistent with those of phase 1 (i.e., perceived risk did not moderate the relationship between the label condition and participant food choice, see Figure 2).

## 4. Discussion

Contrary to the study hypotheses, neither the GMO free nor the contains GMOs labels significantly impacted participants’ self-reported food choice in both phases of our study. Additionally, interaction models indicated that even when participants believed GMOs to be dangerous and attended to a label indicating the presence of GMOs, they still selected such products. This finding indicates that attitude alone does not predict food selection behavior in this context.

A significant amount of work has demonstrated that attitudes are often strongly related to consumer behavior [16,17,34,35]. However, little research has probed how attitudes toward GMOs relate to food choices, and how labels indicating either the presence or absence of GMOs differentially impact consumers’ product evaluations. Previous research has demonstrated both that consumers, on average, hold negative attitudes towards GMOs [8,11] and that “free of” labels can exert different impacts on consumer behavior than “contains” labels [34,35]. Research by Lefebvre et al. [36] found that the inclusion of GM labels stating the presence of modification reduced consumers’ purchase intentions and willingness to pay when compared to non-labeled products. Furthermore, consumers were more averse to animal products containing a GM label than vegetables, indicating a reduced acceptance of genetic modification for specific food types [36]. Alternatively, research by Schouteten et al. [37] found that consumers reacted similarly to products that did not contain a GMO label or contained a free of GMO label, indicating that consumers may assume that non-labeled products are GMO free. Accordingly, the present studies explored how these two types of GMO labels (contains GMOs and GMO free), and how individuals’ attitudes about the potential danger of GMOs, impact consumers’ food choice for a variety of food products. While eye-tracking analysis in the present studies showed that participants attended to GMO free and contains GMOs labels, the presence of these labels did not significantly predict food choice. Perhaps even more surprisingly, one’s attitudes regarding the “danger” GMOs may pose to their health did not play a significant role in the food choice for a product labeled as either being free of GMOs or containing them. This finding indicates an attitude–behavior gap in which self-stated attitudes about GMOs serve as poor predictors of subsequent GMO-related behaviors.

While this attitude–behavior gap may benefit companies who produce GM products, it presents a challenge for researchers, practitioners, and policy makers attempting to understand how consumers perceive and act on risks related to food choices. Additionally, it lends support to the work of Bovay and Alston [38], who assert that the upcoming mandatory GM labeling law in the U.S. will likely be a costly addition to food products and will receive limited attention/use by consumers. In fact, a recent study examining the impact of mandatory bioengineered (BE) labeling used in Vermont indicates that while the label did serve as a useful information source for some consumers, the majority (~66%) did not notice the BE label, limiting its effectiveness [39]. Moreover, the diversity of methodologies and populations studied in previous literature further complicates the identification of factors that might be influencing the observed non-concordance between attitudes and behavior [14,16,24]. For example, previous research has shown that the degree to which self-stated attitudes predict food choice behavior differs depending on whether the food being considered is “health-promoting” or “health-compromising” [14]. This suggests that characteristics such as a food’s healthfulness may impact whether an attitude–behavior gap is present. However, the current research found that even when the health-related characteristics (and all other aspects) of labeled foods were identical (i.e., the foods labeled as containing and not containing GMOs were kept constant between conditions, as in phase 2), there were no significant differences by condition in food choice. Therefore, although, in some cases, food healthfulness might moderate the attitude–behavior gap, in other cases, the gap may persist, regardless of the type of food being assessed, suggesting that more influential predictors than healthfulness exist. In the case of the current work, it is likely that other informational cues, such as the product’s image, health claims, nutritional profile, or price, took precedence over the perceived healthfulness in food choice.

### 4.1. Strengths and Limitations

These studies have several key strengths. First, this paper examines a public health policy-relevant question that will have a significant impact on the food environment, and which has previously been underexplored. Second, eye-tracking data provided objective evidence that participants viewed GMO labels, allowing researchers to confirm that participants were, indeed, attending to the target information. Third, the two studies tested situations that were more externally valid (phase 1) and offered direct comparisons controlling for all other product characteristics (phase 2). The first phase speaks to how foods might be presented in an actual grocery store wherein the same foods that receive contains GMOs labels would not also be labeled as GMO free, while the second phase explored a question that could not be answered in a naturally-occurring food purchasing setting (i.e., what role does the specific food item relative to the GMO label play in driving decision-making?) by labeling the very same products with contains GMOs and GMO free labels, depending on participants’ randomly-assigned study condition.

The current work also has limitations. Although there were over 400 participants in the present study, the sample size did not provide adequate power to detect small effects. It is possible that these labels could have an effect of small, but meaningful magnitude on food purchasing decisions and that this effect was undetected here (although the effect sizes obtained in phases 1 and 2, and reported in Table 2 and Table 5, respectively, suggest that the labels’ effects were near zero). Additionally, it is difficult to determine whether a participant’s food choice as presented in the study would reflect real-world grocery shopping behaviors, indicating that food choice in the present studies may better represent food purchasing in an online—rather than a brick-and-mortar—setting, for example. It is also important to conduct future research with other study populations and to consider additional socio-demographic information, such as family income, field of study/occupation, residential location, etc.

Lastly, it would be helpful to better understand whether individuals’ perceptions of healthfulness are altered by the presence or absence of the “free of” label, the GMO label, or both. Research indicates that when consumers are presented with a “free of” label, their evaluations of the healthfulness of that product increase, irrespective of the actual healthfulness of the product [40]. This indicates that the “free of” label may have a health-halo where the inclusion of the label leads consumers to believe a product is more healthful than a non-labeled alternatives [40]. Additionally, research indicates that consumers perceive GMOs themselves to be less healthful than non-modified alternatives [41]. Future work should strive to determine whether perceptions of healthfulness related to “free of” labeling, GMO labeling, or both influence consumers’ food choices.

Measuring perceived healthfulness in future studies would allow researchers to more concretely discern the impact of heuristic judgments about certain foods (e.g., “fruit is good for my health”) from potential perceptual adjustments related to GMO labeling (e.g., “GMO free fruit is better for my health” or “fruit that contain GMOs is not as good for my health”).

### 4.2. Policy Implications

Lastly, this research has timely policy implications. The recent escalation of national concern about GMOs prompted governmental action in the United States, leading to the passing of Public Law 114–216, which will require (as of 1 January 2022) GM foods to include a “bioengineered” label [42]. By investigating the relationship between consumer attitudes toward GMOs and choice of items with GMO labels at a time when the term GMO was widely recognized [43], this work offers a useful comparison for studies addressing similar questions upon adoption (in 2022) of the “bioengineered” labels and, beyond 2022, when the new terminology has reached a similar degree of recognition as GMOs among consumers. Observing whether the “bioengineered” rebranding presents differently than GM in the relationship between consumer attitudes and purchase intentions is a compelling question for researchers attempting to understand consumer responses in health and marketing fields. This study lends support to the predictions of Bovay and Alston [38], specifically that consumers may respond with indifference to the bioengineered labels. Should this be the case, the costs of mandatory labeling may outweigh the benefits, at least when considering the impact on food choice.

### 4.3. Implications and Future Directions

At the time of data collection (i.e., 2015/2016), GMOs featured prominently in national discussions regarding food safety [44,45,46]. Attitudes toward GM food products are varied, easily changeable, and complex; however, research indicates that the percent of consumers opposed to GMOs is slowly rising. The number of consumers who believe GM products are harmful to one’s health and wish to avoid them entirely rose by 10% from 2016 to 2018 [47]. The results reported here suggest that despite the media attention and consumer negativity toward GMOs, consumers’ food choices may not be affected by such labeling. In anticipation of the forthcoming ‘bioengineered’ labels, the present work can serve as a useful benchmark for comparative analyses.

The present findings also have implications for the global issue of health safety communication. Participants’ lack of responsiveness to labels indicating the presence (or absence) of something many reported as being dangerous to their health speaks to the challenge of health messaging in general. It could be that other factors outweigh avoidance of the perceived danger of GMOs (e.g., taste, price, calories, nutrient content, etc.) or that simply labeling either the presence or absence of that perceived danger (GMOs) did not act as a strong enough cognitive cue to alter behavior (although the current study did confirm that the labels were viewed when present). Future research exploring potential mediators of the attitude–behavior relationship, such as perceived healthfulness or personality differences, would be beneficial. While factors predicting health-related behaviors are diverse and likely dependent on the type of decision and health concern in question, uncovering personal and situational predictors in contexts such as food choice may produce transferable insights useful in various health communication domains.

## 5. Conclusions

While self-stated attitudes tend to be good predictors of behavior, a growing body of research suggests that the relationship between attitude and behavior may be more complex in certain situations [48,49]. The current work showed that labels indicating either the presence or absence of GMOs were insufficient for swaying consumers’ food choices, regardless of attitudes about the potential danger of consuming GMOs. Future research should explore whether similar trends are found in consumer attitudes and behaviors related to the “bioengineered” labels forthcoming in the U.S. market. Additionally, further work identifying factors underlying observed attitude–behavior gaps in health-related realms will help identify creative solutions to bring alignment to the perplexing situations where consumers’ attitudes communicate one thing and their behaviors show another.

## Figures and Tables

**Table 1:** Participant demographic characteristics.

Survey Participant Demographics (*n* = 434)		
	Phase 1	Phase 2
Gender (% Female)	62%	61%
Mean Age (in years)	18.83 (*D* = 1.19)	19.04 (*SD* = 2.05)
Race/Ethnicity (% White)	84.9%	82.1%
Race/Ethnicity (% Hispanic)	14.6%	17.1%
Race/Ethnicity (% Black)	3.6%	1.8%
Race/Ethnicity (% Other)	11.3%	15.7%

**Table 2:** Phase 1 linear regression predicting the number of foods selected.

Predictor	*b*	*B* 95% CI [LL, UL]	*Beta*	*Beta* 95% CI [LL, UL]	*sr* ^2^	*sr*^2^ 95% CI [LL, UL]	*r*	Fit
(Intercept)	34.94	[32.86, 37.02]						
GMO free	0.31	[−2.63, 3.26]	0.02	[−0.14, 0.17]	0.00	[−0.00, 0.00]	0.09	
Contains GMO	−3.05	[−6.08, −0.03]	−0.16	[−0.32, −0.00]	0.02	[−0.02, 0.06]	−0.17	
								*R^2^* = 0.028
								95% CI [0.00, 0.08]

*Note.* A significant *b*-weight indicates that the beta-weight and semi-partial correlation are also significant. *b* represents unstandardized regression weights. *beta* indicates the standardized regression weights. *sr^2^* represents the semi-partial correlation squared. *r* represents the zero-order correlation. *LL* and *UL* indicate the lower and upper limits of a confidence interval, respectively. No test reached the Bonferroni-adjusted level of statistical significance (*p* < 0.017). Genetically modified organism (GMO) free = 32 of 64 products in this condition were labeled GMO free; contains GMOs = 32 of 64 products (those not labeled GMO free in the GMO free condition) in this condition were labeled contains GMOs; for sex, 0 = Male and 1 = Female; for ethnicity, 0 = Not Hispanic/Latina/of Spanish Origin and 1 = Hispanic/Latina/of Spanish Origin; and for race, 0 = White and 1 = Non-White.

**Table 3:** Phase 1 linear regression predicting the number of foods selected; demographics included.

Predictor	*b (se)*	*Beta*	*Beta* 95% CI [LL, UL]	*sr* ^2^	*sr*^2^ 95% CI [LL, UL]	*r*	Fit
(Intercept)	43.31 (11.2)						
GMO free	0.20 (1.56)	0.01	[−0.15, 0.17]	0.00	[−0.00, 0.00]	0.08	
Contains GMOs	−2.83 (1.61)	−0.15	[−0.31, 0.02]	0.02	[−0.02, 0.05]	−0.17	
Age	−0.22 (0.55)	−0.03	[−0.18, 0.12]	0.00	[−0.01, 0.01]	−0.03	
Sex	−0.96 (1.40)	−0.05	[−0.20, 0.10]	0.00	[−0.01, 0.02]	−0.09	
Ethnicity	−2.02 (1.18)	−0.13	[−0.27, 0.02]	0.02	[−0.02, 0.05]	−0.13	
Race	−0.07 (0.17)	−0.03	[−0.18, 0.11]	0.00	[−0.01, 0.01]	−0.01	
							*R*^2^ = 0.048
							95% CI [0.00, 0.09]

*Note.* A significant b-weight indicates that the beta-weight and semi-partial correlation are also significant. b represents unstandardized regression weights. *beta* indicates the standardized regression weights. sr^2^ represents the semi-partial correlation squared. r represents the zero-order correlation. LL and UL indicate the lower and upper limits of a 95% confidence interval, respectively. No test reached the Bonferroni-adjusted level of statistical significance (*p* < 0.017). GMO free = 32 of 64 products in this condition were labeled GMO free; contains GMOs = 32 of 64 products (those not labeled GMO free in the GMO free condition) in this condition were labeled contains GMOs; for sex, 0 = Male and 1 = Female; for ethnicity, 0 = Not Hispanic/Latina/of Spanish Origin and 1 = Hispanic/Latina/of Spanish Origin; and for race, 0 = White and 1 = Non-White.

**Table 4:** Mean number of food items selected per condition.

Condition	Foods Selected (of 64): Phase 1	Foods Selected (of 64): Phase 2
GMO free	35.26	36.33
Contains GMOs	31.89	36.72
No label (control)	34.94	37.69

Note: GMO = genetically modified organism

**Table 5:** Phase 2 linear regression predicting the number of foods selected.

Predictor	*b*	*b* 95% CI [LL, UL]	*Beta*	*Beta* 95% CI [LL, UL]	*sr* ^2^	*sr*^2^ 95% CI [LL, UL]	*r*	Fit
(Intercept)	37.69	[35.11, 40.27]						
GMO free	−1.36	[−4.71, 1.99]	−0.07	[−0.25, 0.10]	0.00	[−0.01, 0.02]	−0.03	
Contains GMOs	−0.97	[−3.94, 2.00]	−0.06	[−0.23, 0.12]	0.00	[−0.01, 0.01]	−0.01	
								*R*^2^ = 0.003
								95% CI [0.00, 0.02]

*Note.* A significant *b*-weight indicates that the beta-weight and semi-partial correlation are also significant. *b* represents unstandardized regression weights. *beta* indicates the standardized regression weights. *sr^2^* represents the semi-partial correlation squared. *r* represents the zero-order correlation. *LL* and *UL* indicate the lower and upper limits of a 95% confidence interval, respectively. No test reached the Bonferroni-adjusted level of statistical significance (*p* < 0.017). GMO free = 32 of 64 products in this condition were labeled GMO free; contains GMOs = 32 of 64 products (those not labeled GMO free in the GMO free condition) in this condition were labeled contains GMOs; for sex, 0 = Male and 1 = Female; for ethnicity, 0 = Not Hispanic/Latina/of Spanish Origin and 1 = Hispanic/Latina/of Spanish Origin; and for race, 0 = White and 1 = Non-White.

**Table 6:** Phase 2 linear regression predicting the number of foods selected; demographics included.

Predictor	*b (se)*	*beta*	*Beta* 95% CI [LL, UL]	*sr* ^2^	*sr*^2^ 95% CI [LL, UL]	*r*	Fit
(Intercept)	51.85 (5.98)						
GMO free	−0.95 (1.73)	−0.05	[−0.23, 0.13]	0.00	[−0.01, 0.01]	−0.04	
Contains GMOs	−0.44 (1.53)	−0.03	[−0.20, 0.15]	0.00	[−0.00, 0.01]	0.01	
Age	−0.53 (0.28)	−0.13	[−0.26, 0.01]	0.02	[−0.02, 0.05]	−0.11	
Sex	−2.41 (1.18)	−0.14	[−0.27, 0.00]	0.02	[−0.02, 0.05]	−0.12	
Ethnicity	−0.31 (0.54)	−0.04	[−0.17, 0.09]	0.00	[−0.01, 0.01]	−0.06	
Race	−0.04 (0.15)	−0.02	[−0.15, 0.11]	0.00	[−0.00, 0.00]	−0.02	
							*R*^2^ = 0.036
							95% CI [0.00, 0.07]

*Note.* A significant *b*-weight indicates that the beta-weight and semi-partial correlation are also significant. *b* represents unstandardized regression weights. *beta* indicates the standardized regression weights. *sr^2^* represents the semi-partial correlation squared. *r* represents the zero-order correlation. *LL* and *UL* indicate the lower and upper limits of a 95% confidence interval, respectively. No test reached the Bonferroni-adjusted level of statistical significance (*p* < 0.017). GMO free = 32 of 64 products in this condition were labeled GMO free; contains GMOs = 32 of 64 products (the *same* foods labeled GMO free in the GMO free condition) in this condition were labeled contains GMOs; for sex, 0 = Male and 1 = Female; for ethnicity, 0 = Not Hispanic/Latina/of Spanish Origin and 1 = Hispanic/Latina/of Spanish Origin; and for race, 0 = White and 1 = Non-White.

**Figure A1:**
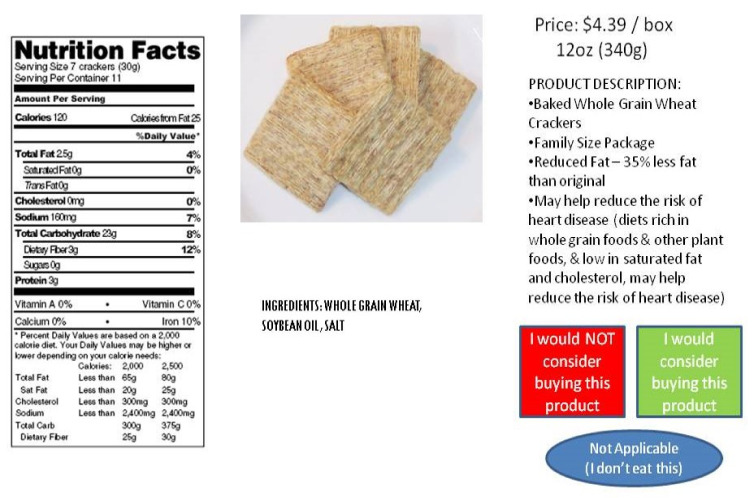
Sample product selection task for those in the no GMO label (control) condition.

**Figure A2:**
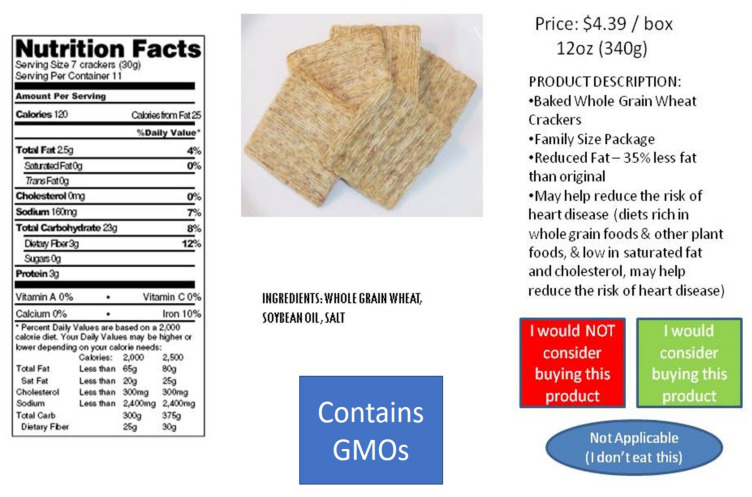
Sample product selection task for those in the contains GMOs condition.

**Figure A3:**
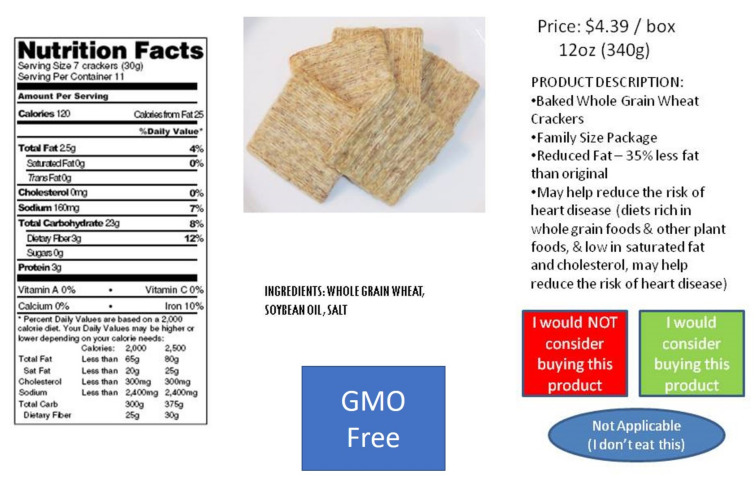
Sample product selection task for those in the GMO free condition.

## Data Availability

The data presented in this study are openly available in the Open Science Framework at https://doi.org/10.17605/OSF.IO/CP739.

## Appendix A

After running the full MLR model, which included responses for all 64 food items, the models were run again, this time only including the 32 food items that had labels pertaining to GMOs in both phases 1 and 2. Consistent with Schouteten [37], no significant differences were found between the GMO free condition and the control condition (see Table A1). Additionally, when comparing the contains GMOs condition to the control condition, no significant differences were identified (see Table A1). Taken together, the results indicate that the GMO labels themselves did not have a significant impact on participants’ food choice.

**Table A1:** Mean number of foods selected per condition.

Condition	Foods Selected (of 64 Foods) Phase 1	Foods Selected (of 32 Foods with Labels in GMO Conditions) Phase 1	Foods Selected (of 64 Foods) Phase 2	Foods Selected (of 32 Foods with Labels in GMO Conditions) Phase 2
GMO free	35.26	17.43	36.33	18.33
Contains GMOs	31.89	15.81	36.72	18.21
No label (control)	34.94	17.51 (Free)/17.43 (Contains)	37.69	18.50

Note: GMO = genetically modified organism

## Appendix C

**Table A2:** Food items (*n* = 64) with their Phase 1 and Phase 2 GMO labels by experimental condition.

		Phase 1			Phase 2		
	Item	GMO Free	Contains GMOs	Control	GMO Free	Contains GMOs	Control
1	Crackers (Whole Wheat)	Free ^a^	-- ^b^	--	Free	Contains ^c^	--
2	Crackers (Soda)	--	Contains	--	--	--	--
3	Crackers (Multigrain)	Free	--	--	Free	Contains	--
4	Crackers (Nut Flour)	--	Contains	--	--	--	--
5	Peanuts (Salted, No Shell)	Free	--	--	Free	Contains	--
6	Peanuts (Honey Roasted, No Shell)	--	Contains	--	--	--	--
7	Almonds (Cinnamon)	Free	--	--	Free	Contains	--
8	Almonds (Plain, Salted)	--	Contains	--	--	--	--
9	Cereal (Wheat Biscuits, No Frosting)	Free	--	--	Free	Contains	--
10	Cereal (Wheat Flakes)	--	Contains	--	--	--	--
11	Cereal (Corn Flakes, Frosted)	Free	--	--	Free	Contains	--
12	Cereal (Oat Cereal, Honey Nut Flavored)	--	Contains	--	--	--	--
13	Cereal (Wheat Biscuits, Frosted)	Free	--	--	Free	Contains	--
14	Cookies (Fig Bars)	--	Contains	--	--	--	--
15	Cookies (Chocolate Chip, Crunchy)	Free	--	--	Free	Contains	--
16	Cookies (Chocolate Chip, Chewy)	--	Contains	--	--	--	--
17	Cookies (Chocolate Chip, Crunchy, Miniature)	Free	--	--	Free	Contains	--
18	Cookies (Chocolate Wafer Sandwiches with Vanilla Crème)	--	Contains	--	--	--	--
19	Chips (Multigrain)	Free	--	--	Free	Contains	--
20	Chips (Potato)	--	Contains	--	--	--	--
21	Chips (Potato, Stackable)	Free	--	--	Free	Contains	--
22	Chips (Corn)	--	Contains	--	--	--	--
23	Pretzels	Free	--	--	Free	Contains	--
24	Rice Cakes	--	Contains	--	--	--	--
25	Cheese Puffs	Free	--	--	Free	Contains	--
26	Popcorn	--	Contains	--	--	--	--
27	Fruit Cocktail (in 100% Juice)	Free	--	--	Free	Contains	--
28	Fruit Cocktail (in Syrup)	--	Contains	--	--	--	--
29	Apple Sauce	Free	--	--	Free	Contains	--
30	Pears (Canned, No Sugar Added)	--	Contains	--	--	--	--
31	Strawberries (Frozen)	Free	--	--	Free	Contains	--
32	Blueberries (Frozen)	--	Contains	--	--	--	--
33	Raspberries (Frozen)	Free	--	--	Free	Contains	--
34	Mixed Fruit (Frozen)	--	Contains	--	--	--	--
35	Pizza (Large, Frozen, Cheese)	Free	--	--	Free	Contains	--
36	Pizza (Small, Frozen, Cheese)	--	Contains	--	--	--	--
37	Pizza (Bite-sized, Frozen, Cheese)	Free	--	--	Free	Contains	--
38	Pizza (Pockets, Frozen, Cheese)	--	Contains	--	--	--	--
39	Pizza (Focaccia, Frozen, Cheese)	Free	--	--	Free	Contains	--
40	Yogurt (Plain)	--	Contains	--	--	--	--
41	Yogurt (Peach)	Free	--	--	Free	Contains	--
42	Yogurt (Blueberry)	--	Contains	--	--	--	--
43	Yogurt (Strawberry)	Free	--	--	Free	Contains	--
44	Ice Cream (Vanilla, Low Fat)	--	Contains	--	--	--	--
45	Ice Cream (Vanilla, Full Fat)	Free	--	--	Free	Contains	--
46	Ice Cream (Chocolate Chip Cookie Dough)	--	Contains	--	--	--	--
47	Ice Cream (Mint Chocolate Cookie)	Free	--	--	Free	Contains	--
48	Turkey (Smoked)	--	Contains	--	--	--	--
49	Turkey (Oven Roasted)	Free	--	--	Free	Contains	--
50	Ham (Baked)	--	Contains	--	--	--	--
51	Ham (Honey)	Free	--	--	Free	Contains	--
52	Soup (Chicken Noodle, Classic, Canned)	--	Contains	--	--	--	--
53	Soup (Chicken Noodle, Lower Sodium, Canned)	Free	--	--	Free	Contains	--
54	Soup (Vegetable, Boxed)	--	Contains	--	--	--	--
55	Soup (Vegetable, Canned)	Free	--	--	Free	Contains	--
56	Corn (Low Sodium, Canned)	--	Contains	--	--	--	--
57	Corn (Regular, Canned)	Free	--	--	Free	Contains	--
58	Peas (Regular, Canned)	--	Contains	--	--	--	--
59	Mixed Vegetables (Regular, Canned)	Free	--	--	Free	Contains	--
60	Peas (Regular, Frozen)	--	Contains	--	--	--	--
61	Mixed Vegetables (Regular, Frozen)	Free	--	--	Free	Contains	--
62	Corn (Regular, Frozen)	--	Contains	--	--	--	--
63	Corn (Organic, Frozen)	Free	--	--	Free	Contains	--
64	Peas (Organic, Frozen)	--	Contains	--	--	--	--

Notes: GMO = Genetically Modified Organism. ^a^ Free, labels said “GMO Free”; ^b^ --, indicates no GMO labels were present; ^c^ contains, labels said “Contains GMOs”.
